# Demographics of Vaccine Hesitancy in Chandigarh, India

**DOI:** 10.3389/fmed.2020.585579

**Published:** 2021-01-15

**Authors:** Abram L. Wagner, Abigail R. Shotwell, Matthew L. Boulton, Bradley F. Carlson, Joseph L. Mathew

**Affiliations:** ^1^Department of Epidemiology, School of Public Health, University of Michigan, Ann Arbor, MI, United States; ^2^Division of Infectious Disease, Department of Internal Medicine, University of Michigan Medical School, Ann Arbor, MI, United States; ^3^Department of Pediatrics, Advanced Pediatrics Centre, Postgraduate Institute of Medical Education and Research Chandigarh, Chandigarh, India

**Keywords:** vaccine hesitancy, India, education, religion, caste, vaccination coverage

## Abstract

The impact of vaccine hesitancy on childhood immunization in low- and middle-income countries remains largely uncharacterized. This study describes the sociodemographic patterns of vaccine hesitancy in Chandigarh, India. Mothers of children <5 years old were sampled from a two-stage cluster, systematic sample based on Anganwadi child care centers in Chandigarh. Vaccine hesitancy was measured using a 10-item Vaccine Hesitancy Scale, which was dichotomized. A multivariable logistic regression assessed the association between socioeconomic factors and vaccine hesitancy score. Among 305 mothers, >97% of mothers thought childhood vaccines were important, effective, and were a good way to protect against disease. However, many preferred their child to receive fewer co-administered vaccines (69%), and were concerned about side effects (39%). Compared to the “other caste” group, scheduled castes or scheduled tribes had 3.48 times greater odds of vaccine hesitancy (95% CI: 1.52, 7.99). Those with a high school education had 0.10 times the odds of vaccine hesitancy compared to those with less education (95% CI: 0.02, 0.61). Finally, those having more antenatal care visits were less vaccine hesitant (≥4 vs. <4 visits OR: 0.028, 95% CI: 0.1, 0.76). As India adds more vaccines to its Universal Immunization Program, consideration should be given to addressing maternal concerns about vaccination, in particular about adverse events and co-administration of multiple vaccines.

## Introduction

Childhood vaccination programs contributed to major reductions in global morbidity and mortality in children under 5 years (1). Population-level benefits of childhood immunization can only be achieved if a high proportion of children are immunized. Suboptimal vaccine uptake has historically been thought to largely result from barriers to vaccine access (2). More recently, though, vaccine hesitancy has been recognized as an important emerging risk factor for non-vaccination (3), and was listed as one of the World Health Organization (WHO)'s Ten Threats to Global Health in 2019 (4). In response to the growing concerns about increased vaccine hesitancy and lack of a commonly agreed upon and standardized definition, the World Health Organization Strategic Advisory Group of Experts on Immunization (SAGE) Working Group on Vaccine Hesitancy defined vaccine hesitancy as the “delay in acceptance or refusal of vaccination despite availability of vaccination services. Vaccine hesitancy is complex and context specific, varying across time, place, and vaccines. It is influenced by factors such as complacency, convenience, and confidence” (5).

Reductions in vaccine coverage related to increasing vaccine hesitancy can lead to endemic transmission and outbreaks of preventable diseases. In northern Nigeria, fears of contamination of polio vaccines with antifertility drugs and HIV resulted in a boycott of the vaccine in 2003 and 2004 (6). The subsequent reduction in polio vaccine coverage gave rise to transmission of polio from Nigeria to several countries that had previously been declared polio-free (7). In the United States, vaccine hesitancy among subpopulations contributed to outbreaks of vaccine preventable diseases, including a 2011 outbreak of measles in Minnesota that was linked to concerns among the Somali-American community that vaccination against measles was associated with autism (8). And more recently in the United States, in 2019 there were 1,090 cases of measles in those ≥1 year, with 66% unvaccinated (9). Most vaccine hesitancy research has been conducted in high income countries, but understanding the prevalence of factors related to vaccine hesitancy globally, regardless of a country's level of economic development, is important for sustainably increasing levels of immunization coverage and addressing the health burden of vaccine preventable diseases globally.

India has a large number of unvaccinated and undervaccinated children. As of 2019, India is home to the world's second largest population of infants without the first dose of diphtheria-tetanus-pertussis vaccine (10). Official country estimates show that over 90% of children have been administered Bacillus Calmette-Guérin, the third dose of diphtheria-tetanus-pertussis vaccine, the third dose of polio vaccine, and the measles vaccine. Non-official coverage estimates show lower coverage, but are from earlier years (11). For example from a survey in 2012–13, only 59% of children 12–48 months were fully vaccinated (12). In India, studies have found that knowledge of the importance of vaccines, awareness of logistics of vaccination, beliefs in vaccine effectiveness, perceived risk of vaccine-preventable diseases, and concerns of adverse effects can impact vaccine uptake in specific populations (13–15). Previous research in India examined attitudes toward vaccines (16–20) and the relationship between attitudes and vaccine uptake (13–15), although none of these studies used the WHO SAGE Vaccine Hesitancy Scale (21) to measure vaccine hesitancy nor have prior studies elucidated the impact of sociodemographic characteristics on vaccine hesitancy in India. Additionally, India has recently initiated, or is in the process of rolling out several pediatric vaccines into its Universal Immunization Program, including *Haemophilus influenzae* type b vaccine (Hib), measles-rubella vaccine (MR), rotavirus vaccine, and pneumococcal conjugate vaccines (PCV) (22), which likely will necessitate more co-administration (i.e., simultaneous administration) of vaccines at a given clinic visit than what was done previously. The National Health Portal of India maintains a list of currently recommended vaccines (23).

Greater understanding is needed about the prevalence of vaccine hesitancy in communities in India and associations with demographic characteristics. This study aimed to fill this gap by exploring the relationship between sociodemographic factors and vaccine hesitancy in Chandigarh, India, and how that manifests in the context of full vaccination status utilizing the WHO SAGE Vaccine Hesitancy Scale.

## Materials and Methods

### Study Population

Chandigarh is a city in north India that serves as the capital for two different states. This study took place between June 2017 and June 2018. This study was embedded in a larger project whose aim was to characterize measles antibody levels. In this study, we wanted 450 individuals 21–40 years old in order to estimate zero-positivity in adults with a confidence interval of ±0.044 with an estimated proportion of 0.90. This vaccine hesitancy project started data collection after the original measles study began, and so the individuals involve represent a convenience sample of the original study population.

Households were selected through a multi-stage selection technique. The first stage was the catchment area around an Anganwadi, a publicly funded child care center. Chandigarh contains 510 Anganwadi centers, with each roughly serving a population of about 3,000. We selected 30 Anganwadis from a list. At each Anganwadi, workers maintain a list of households in their catchment area in paper registers. Prior to going to each Anganwadi, the research staff were given a list of random numbers, which corresponded to the pages of the Anganwadi household registers. In this way, research staff put together a list of potential households to contact. If a household was ineligible, refused, or did not have any person available, we proceeded to the next household on the list. To be included in the study, potential participants had to be a mother of a child ≤5 years of age who was herself 20–40 years old. Participants were verbally administered the questionnaire in Hindi.

An abbreviated study protocol is available as a Supplementary File. A dataset limited to the analyzed variables, questionnaire, and code are all available on figshare: https://doi.org/10.6084/m9.figshare.13247363.v1.

### Derived Variables

**Figure 1:**
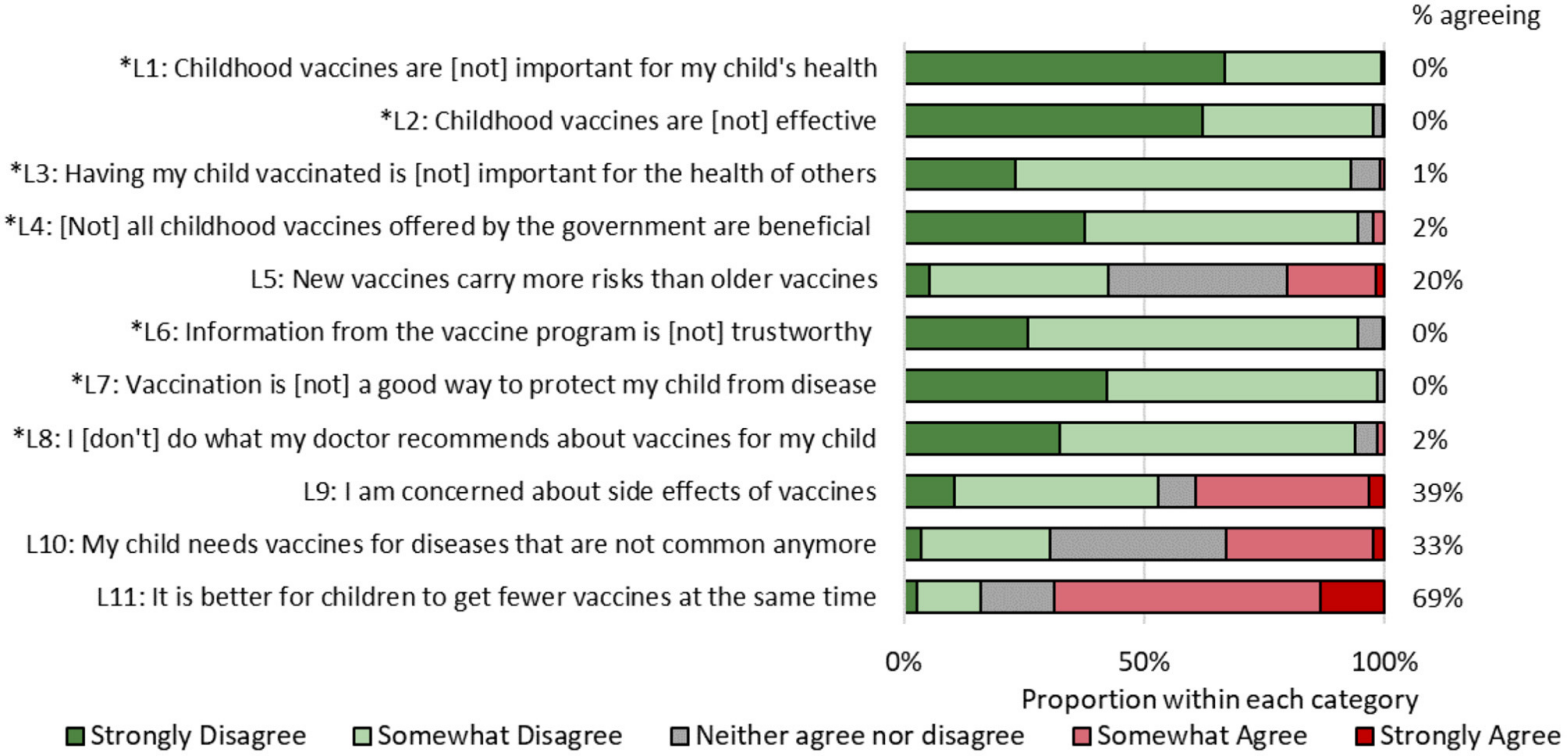
Responses to vaccine hesitancy scale, Chandigarh, India, 2017. Asterisked items have been reverse-coded.

The WHO SAGE Working Group on Vaccine Hesitancy developed a 10-item Vaccine Hesitancy Scale (VHS) (21). This survey has previously been used in Canada (24), China (25), Guatemala (26), and Ethiopia (27). Questions from this scale are shown in Figure 1. Based on past research (28), it is clear that many parents have negative attitudes toward vaccine co-administration, hence we added an item about vaccine co-administration to the VHS. Items were assessed on a 5-point Likert scale, from “strongly disagree” to “strongly agree.” The responses to items L1–L4 and L6–L8 were flipped so that higher responses on the Likert scale were indicative of greater vaccine hesitancy. All items had acceptable internal reliability (standardized Crohnbach's α = 0.73, *N* = 305). We constructed a dichotomous vaccine hesitancy variable by first summing all responses from the original ten items of the VHS, each which ranged from 1 to 5 so that the total score could range from 10 to 50. We then created a cut-off point of 25, which represented the top 10% most hesitant individuals in the dataset.

Full vaccination was defined as receipt of one dose of bacillus Calmette-Guérin (BCG), three doses diphtheria-tetanus-pertussis vaccine (DTP), three doses polio vaccine (IPV or OPV), and one dose of measles-containing vaccine (MCV) among children at least 1 year old. Children with at least one of these doses missing were categorized as not fully vaccinated. Almost all women (99%) had vaccination cards for their children. Participants were first asked about what vaccines their child received, and these were verified in the vaccination card.

Participants also responded to socioeconomic questions. Participant age and age of youngest child were both categorized into tertiles. Responses to some other variables were condensed due to low numbers in certain groups. Caste was defined as follows: scheduled caste and scheduled tribe were combined into one category, individuals listing “unknown” (*n* = 2) were collapsed into the Other Backward Caste (OBC) category, and the rest were categorized as others. Historically, individuals in scheduled castes and scheduled tribes have been the most disadvantaged, and below those in the Other Backward, who are below the most historically advantaged “Others” group. Monthly household income was dichotomized based on 10,000 Rs (Indian Rupees) (equivalent to $143). Delivery location was divided into those who had an institutional birth vs. those with a non-institutional birth (regardless of whether they had a skilled or unskilled attendant). Antenatal care was divided between those with ≤3 visits and those with ≥4 visits. Distance from vaccination site was dichotomized as those living <15 min from vaccination site and those living ≥15 min away.

### Statistical Analysis

We describe the proportion who were vaccine hesitant across different demographic groups. The significance of the association between socioeconomic groups and vaccine hesitancy was assessed through a multivariable logistic regression model with output odds ratios (ORs) and 95% confidence intervals (CI). All covariates (age, caste, religion, monthly income, education, number of children in household, distance from vaccination site, youngest child's sex, youngest child's age, delivery place, number of antenatal care visits) were included in the model based on an *a priori* hypothesis.

We ran three subsequent logistic regression models, where vaccine hesitancy was the predictor, and full vaccination of child, ever refused a vaccine, and ever delayed a vaccine were the three outcomes. Inverse probability of treatment weights were constructed to account for confounding (29). Briefly, the previous model was used to calculate predicted probabilities of vaccine hesitancy status. Stabilized weights were constructed as the probability that an individual would be assigned their observed vaccine hesitancy status with no covariates in the model over the probability they would be assigned their status with all covariates in the model. The model for full vaccination status was limited to children at least 1 year of age.

All analyses accounted for clustering of data due to interview location (Anganwadi health center) using survey procedures in the SAS version 9.4 (SAS Institute, Cary, NC). An α level of 0.05 was considered the boundary of significance, and 95% confidence intervals were calculated to assess precision of results.

### Ethical Considerations

This study was reviewed by the University of Michigan Health Sciences and Behavioral Sciences Institutional Review Board in Ann Arbor, MI, USA (#HUM00126619) and the Postgraduate Institute of Medical Education and Research in Chandigarh, India (#PGI/IEC/2015/1363).

## Results

Overall, we approached 412 households. A total of 103 households decided not to participate, leaving a total of 309 households with mothers (75%) who were interviewed. Four participants did not respond to all vaccine hesitancy questions and were excluded from all analyses, leaving a final sample size of 305. Supplementary Figure 1 is a flowchart of the selection procedure.

A description of the study population is shown in Table 1. Participant age ranged from 20 to 40 years, with a mean age of 27.9 years; age of the (youngest) child of these mothers ranged from 0.2 to 5.7 years, with a mean age of 3.0 years. Most belonged to “other” category of caste (56%), and the majority were Hindu (83%). The average monthly income was low in the study population; 61% earned <10,000 Rs ($143 USD) each month. Almost half of the women (45%) had two children in their care. A majority of participants lived <15 min from the vaccination site (77%), delivered their (youngest) child at a government institution (92%), and had ≥4 antenatal care visits in the last pregnancy (87%).

**Table 1:** Vaccine hesitancy by demographic characteristics, Chandigarh, India, 2017–2018.

**Demographic factor**		**Count (column %)**	**Vaccine hesitant (row %)**
Overall		305 (100%)	29 (10%)
Participant age (Years)	20–26	106 (37%)	11 (10%)
	27–29	99 (34%)	9 (9%)
	30–40	85 (29%)	9 (11%)
Caste	Scheduled caste or tribe	120 (39%)	20 (17%)
	Backward or unknown caste	16 (5%)	2 (13%)
	Other	169 (55%)	7 (4%)
Religion	Hindu	254 (83%)	21 (8%)
	Sikh	19 (6%)	2 (11%)
	Muslim	32 (10%)	6 (19%)
Household monthly income	Rs <10,000 ($143)	185 (61%)	24 (13%)
	Rs ≥ 10,000 ($143)	120 (39%)	5 (4%)
Participant education	Less than high school	218 (71%)	28 (13%)
	High school or higher	87 (29%)	1 (1%)
Number of children in care	1	85 (28%)	9 (11%)
	2	137 (45%)	10 (7%)
	3	60 (20%)	5 (8%)
	≥4	23 (8%)	5 (22%)
Distance from vaccination site	<15 min	233 (76%)	24 (10%)
	≥15 min	72 (24%)	5 (7%)
Youngest child's sex	Male	151 (50%)	11 (7%)
	Female	153 (50%)	18 (12%)
Age of youngest child	<2.5 years	107 (35%)	11 (10%)
	2.5–3.5 years	92 (30%)	8 (9%)
	>3.5 years	103 (34%)	9 (9%)
Delivery place of youngest child	Institutional	289 (95%)	23 (8%)
	Non-institutional	16 (5%)	6 (38%)
Number of antenatal care visits	<4	39 (13%)	11 (28%)
	≥4	259 (87%)	18 (7%)
Ever delayed a vaccine	No	278 (91%)	22 (8%)
	Yes	27 (9%)	7 (26%)
Ever refused a vaccine	No	280 (92%)	24 (9%)
	Yes	25 (8%)	5 (20%)
Youngest child fully vaccinated^a^	No	56 (21%)	6 (11%)
	Yes	217 (79%)	19 (9%)

a *Limited to children at least 1 year old*.

There was high agreement among participants regarding the benefits of vaccines (Figure 1). None of the mothers responded affirmatively to items L1 [vaccines are (not) important for my child's health] and L7 [vaccination (does not) protect my child from disease]. Mothers expressed the greatest agreement about preferring to have their child receive fewer vaccines at once (69%), being concerned about side effects (39%), not obtaining vaccines for diseases no longer common (33%), and that new vaccines are riskier than older vaccines (20%).

Table 2 shows the results of the multivariable logistic regression of vaccine hesitancy score by sociodemographic factors. Vaccine hesitancy was associated with caste, maternal education, and number of antenatal care visits. Compared to the “other caste” group, scheduled castes or scheduled tribes had 3.48 times greater odds of vaccine hesitancy (95% CI: 1.52, 7.99). Those with a high school education had 0.10 times the odds of vaccine hesitancy compared to those with less education (95% CI: 0.02, 0.61). Finally, those having more antenatal care visits were less vaccine hesitant (≥4 vs. <4 visits OR: 0.028, 95% CI: 0.1, 0.76).

**Table 2:** Multivariable logistic regression results for the outcome of vaccine hesitancy, Chandigarh, India, 2017–2018 (*N* = 281).

**Independent variable**		**OR (95% CI)**	**P^a^**
Participant age (Years)	20–26	ref	0.9177
	27–29	1.06 (0.31, 3.61)	
	30–40	1.23 (0.45, 3.40)	
Caste	Scheduled caste or tribe	3.48 (1.52, 7.99)	0.0045
	Backward or unknown caste	0.89 (0.13, 6.05)	
	Other	ref	
Religion	Hindu	ref	0.0158
	Sikh	7.07 (1.51, 33.10)	
	Muslim	3.16 (0.67, 15.02)	
Household monthly income	Rs <10,000 ($143)	ref	0.5899
	Rs ≥ 0,000 ($143)	0.70 (0.18, 2.68)	
Participant education	Less than high school	ref	0.0139
	High school or higher	0.10 (0.02, 0.61)	
Number of children in care	1	ref	0.3830
	2	0.57 (0.20, 1.57)	
	3	0.43 (0.14, 1.28)	
	≥4	0.74 (0.21, 2.64)	
Distance from vaccination site	<15 min	ref	0.3238
	≥15 min	0.52 (0.14, 1.98)	
Youngest child's sex	Male	ref	0.2215
	Female	2.10 (0.62, 7.04)	
Age of youngest child (Years)	<2.5 years	ref	0.7799
	2.5–3.5 years	0.74 (0.30, 1.81)	
	>3.5 years	0.65 (0.14, 3.02)	
Delivery place of youngest child	Institutional	0.49 (0.11, 2.25)	0.3464
	Non-institutional	ref	
Number of antenatal care visits	<4	ref	0.0146
	≥4	0.28 (0.10, 0.76)	

*CI, confidence interval; OR, odds ratio*.

a *Type 3 Analysis of effects*.

Models of vaccine hesitancy and various other vaccination outcomes are found in Table 3. Approximately 79% of children ≥1 year old were fully vaccinated. The odds of full vaccination were 0.36 times as high among children whose mothers were vaccine hesitant (95% CI: 0.06, 2.17) compared to children whose mothers were not vaccine hesitant. In total, 8% of mothers had refused a vaccine at some point; and mothers who were vaccine hesitant had 4.63 time greater odds of vaccine refusal (95% CI: 0.78, 27.4). About 9% had delayed a vaccine, and vaccine delay was significantly higher among mothers who were vaccine hesitant (OR: 5.80, 95% CI: 1.02, 33.0, *P* = 0.0475).

**Table 3:** Relationship between vaccine hesitancy and three vaccination outcomes, Chandigarh, India, 2017–2018.

	**Full vaccination^a^^,^^b^**	**Ever refused a vaccine^a^**	**Ever delayed a vaccine^a^**
	**OR (95% CI)**	**OR (95% CI)**	**OR (95% CI)**
	***N* = 254**	***N* = 281**	***N* = 281**
Vaccine hesitant	0.36 (0.06, 2.17)	4.63 (0.78, 27.4)	5.80 (1.02, 33.0)

*CI, confidence interval; OR, odds ratio*.

a *Model included a stabilized inverse probability of treatment weight, that was calculated based on participant age, caste, religion, monthly income, education, number of children, distance from vaccination site, child's sex, child's age, institutional delivery, and number of antenatal care visits*.

b *Limited to children at least 1 year old*.

## Discussion

Vaccine hesitancy is currently under-studied in low and middle income countries, and relevant research from India, which has the largest annual birth cohort and administers the greatest number of childhood vaccines globally, are disconcertingly sparse. Given the substantial potential for vaccine hesitancy to lower vaccination coverage and potentiate the spread of highly morbid diseases, further exploration of parental concerns about vaccines in India is clearly warranted. This study found that mothers generally agreed on the benefits of vaccines, although a significant proportion expressed specific concerns about the safety of vaccines, the continued use of vaccines in an era of low incidence of certain vaccine-preventable diseases, the use of new vaccines, and vaccine co-administration. These concerns are particularly noteworthy as India seeks to eliminate certain vaccine-preventable diseases, like measles, while also introducing new vaccines into the Universal Immunization Program as the government expands their recommended vaccination schedule.

Several previous studies have examined vaccine hesitancy in multiple contexts. A 2018 Wellcome Trust study on vaccine hesitancy found that over 95% of Indian parents surveyed believed vaccines to be safe, effective, and important (30). Nonetheless, there is some evidence that non-acceptance of vaccines is emerging as a reason for non-vaccination in India. Among mothers who had a child who had not received at least one vaccine, the proportion indicating lack of acceptance as a reason for not vaccinating their child increased from <10% to over 20% between 1998 and 2007 (13). Given India's large birth cohort—the largest in the world currently—and its status as the country with the second highest number of children without the DTP3 vaccine [2.9 million in 2017 (31)], there is greater need to better understand the potential role of vaccine hesitancy and its impact on population level coverage.

Other studies utilizing the WHO SAGE VHS in Canada (24), China (25), Guatemala (26), and Ethiopia (27) also found an overall positive attitude toward vaccines in their study populations, but with similar expressed concerns regarding new vaccines and serious adverse effects. Additionally, like our study, Shapiro et al. found that education level was significantly associated with vaccine hesitancy (24), and Ren et al. found that lower education was associated with a greater degree of belief that new vaccines carry more risks (25). However, three of these studies (24–26) used factor analysis or some measure of internal consistency to group survey items into components. We chose to examine a dichotomous variable after checking all survey items for internal consistency, which was acceptable, in contrast to the other sites. It will be an interesting area of future research to determine what the local vs. global characteristics of vaccine hesitancy are.

Vaccination status could be associated with a variety of sociodemographic factors. Other studies in the region, including a study of attitudes toward influenza vaccines in Pakistan, found that higher education levels were associated with more positive attitudes toward vaccines (32). Studying the role of caste on vaccination is pertinent (and under-studied) given its long history as a hierarchical social construct in India. Previously, studies using nationwide datasets from India from 2007 and 2008 have shown that compared to individuals in the other (i.e., historically more privileged castes), those in the Other Backward Castes and Scheduled Castes or Scheduled Tribes had lower vaccination coverage (33, 34). Although the relationship between caste and vaccination status could be mediated by access to and availability of health care, our study provides additional evidence that attitudes toward vaccination could impact this relationship as well.

In this study there was a positive relationship between education and vaccine hesitancy, with more educated mothers less likely to be vaccine hesitant. Previous studies conducted in Shanghai, China (25, 28) found a similar relationship. However, several studies in the United States found an opposite relationship, where a more highly educated or affluent individual was more likely to be vaccine hesitant or refuse vaccines (35–37). Overall, the diversity of these findings indicate that there is not a uniform relationship between socioeconomic status and vaccine hesitancy, but this may vary in countries with different economic and socio-cultural contexts. For example, in this study population, Sikh mothers had greater vaccine hesitancy than Hindu mothers, after adjusted for other socioeconomic factors. Future quantitative and qualitative studies can explore reasons why. Continued study of risk factors for vaccine hesitancy in LMICs, where there is a disproportionate burden of vaccine-preventable disease and where most childhood vaccinations are administered, is extremely important.

Number of antenatal care visits point to the role of health care system in forming an individual's opinions about vaccines. Health care workers are highly influential sources of vaccine information. Parents having a strong relationship with a health care provider or the health care profession—through repeat antenatal care visits, for example—can prevent adoption of anti-vaccination attitudes and practices (38). Parents who are able to gain vaccine information from physicians (instead of other parents or the internet) can also lead to parents less concerned about vaccinations (28, 39).

We did not find a significant relationship between vaccine hesitancy and full vaccination status, although vaccine hesitancy has been linked to vaccination status in Ethiopia (27) and the United States (40). The lack of association in our study could have resulted from a small sample size and limited statistical power, or it could reflect a real situation in India, in that parents may have concerns about vaccines, but these concerns do not yet affect their vaccination behaviors, at least for those vaccines that we measured that were on the Universal Immunization Program. These associations could definitely change in the future as patterns of health care, education, and access to social and mass media change.

The on-going COVID-19 pandemic (41) could interplay with vaccine hesitancy, particularly as previous literature has documented increases in vaccine hesitancy in response to pandemics (42). In nearby Pakistan, there has been COVID-19 conspiracy movements which could increase vaccine hesitancy and impact eventual vaccine uptake (43). Although this survey in Chandigarh, India, found relatively muted vaccine hesitancy in the general population, perceptions could rapidly shift with misinformation spread during the COVID-19 pandemic. Addressing problems in adult vaccination in India may be particularly challenging, as most vaccination recommendations focus on children, and the seasonal influenza guidelines focus on adults with chronic disease (44).

### Strengths and Limitations

A strength of this study was the use of the WHO SAGE vaccine hesitancy scale, which provides a standard for comparison purposes in other countries globally. In contrast with previous studies in India, we also asked specific questions about vaccine concerns. However, this study has several limitations including the collection of limited socioeconomic information which could have missed important variables affecting vaccine hesitancy. This study is limited to one city in India and is not generalizable to the entire country. Sampling through Anganwadis could also limit access to more recent migrants into the city. By limiting the sample to mothers at least 20, we have missed younger mothers who may have different vaccination behaviors. Additionally, wealthier individuals may preferentially use private child care centers, although their household information should still have been on the Anganwadi registers. Because this survey was administered face-to-face, it could be subject to social desirability bias or other interviewer effects.

## Conclusions

Although we did not find evidence that vaccine hesitancy was related to full vaccination status, we did find that mothers had specific concerns about vaccine safety, co-administration, new vaccines, and the use of vaccines for low incident diseases. We utilized the SAGE hesitancy scale which is intended to provide a standardized framework for studying hesitancy for future studies in India but also for relevant studies in other countries globally. As India adds more recommended vaccines into the Universal Immunization Program, the government should consider adoption of educational and outreach efforts directed at addressing these parental concerns about vaccines in order to stem vaccine hesitancy while also promoting childhood vaccination.

## Data Availability Statement

The datasets presented in this study can be found in online repositories. The names of the repository/repositories and accession number(s) can be found below: https://doi.org/10.6084/m9.figshare.13247363.v1.

## Ethics Statement

The studies involving human participants were reviewed and approved by the University of Michigan Health Sciences and Behavioral Sciences Institutional Review Board in Ann Arbor, MI, USA (#HUM00126619) and the Postgraduate Institute of Medical Education and Research in Chandigarh, India (#PGI/IEC/2015/1363). The patients/participants provided their written informed consent to participate in this study.

## Author Contributions

AW conceived of the study idea, drafted the manuscript, and supervised the data analyses. AS drafted the manuscript and performed the initial analyses. MB reviewed the manuscript for critical intellectual content. BC helped with data management, and reviewed the manuscript for critical intellectual content. JM supervised data collection and reviewed the manuscript for critical intellectual content. All authors agreed to the final version and act as guarantors of the work.

## Conflict of Interest

The authors declare that the research was conducted in the absence of any commercial or financial relationships that could be construed as a potential conflict of interest.
