# Supplementary material for: Demographics of Vaccine Hesitancy in Chandigarh, India
Source: Front Med (Lausanne). 2021 Jan 15;7:585579. doi: 10.3389/fmed.2020.585579 (PMC7844137; doi:10.3389/fmed.2020.585579)
Supplement: Supplementary file 1 [file Table_1.DOCX]

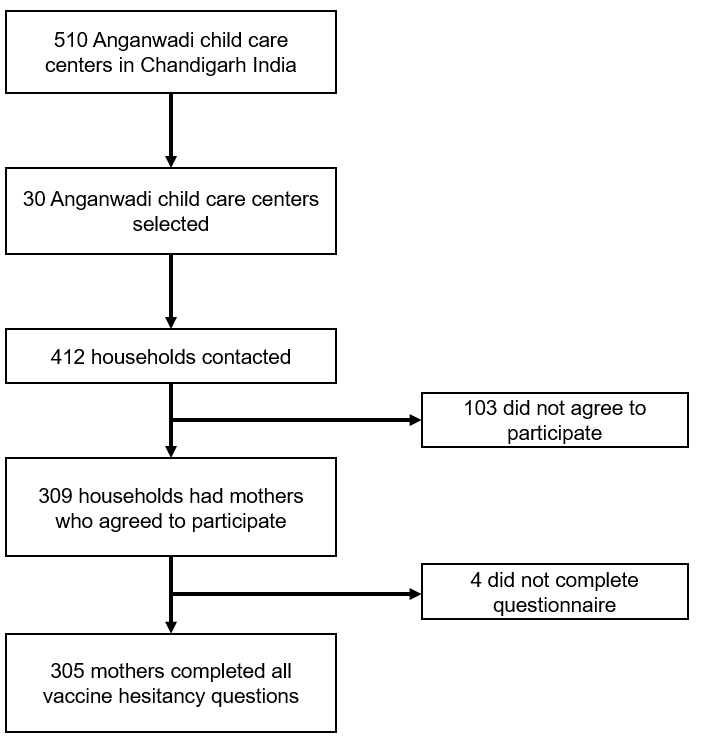


**Supplementary Figure 1.** Selection of mothers into a study of vaccine hesitancy in Chandigarh, India.

More detailed notes about study selection in the larger measles seroprevalence study are included below.

**Study Design**

The design of this population-based study is cross sectional for most participants, with a longitudinal component for infants. This study will consist of one-time participation for all participants over the age of 1 year and 5 visits (birth, 3, 6, 9, and 12 months) for infants, who will be recruited at birth. A questionnaire will be administered by a trained interviewer, and a venipuncture sample will be collected at each study visit. A 2 ml sample would be preferred for all participants, but we will obtain only 1 ml from infants. The process of obtaining informed consent, specimen collection and interview will take approximately 15-25 minutes. We will enroll 1500 study participants from Chandigarh, India during the two years of data collection. Participants will range in age from 0 to 60 years. Both infants (children under age 1) and their mothers will be sampled to permit determination of immunity concordance between those infants and their mothers. Table 1 provides the number of participants to be recruited from the general population, subdivided by age.

Table 1: Sample size by age

| Age range | Number of participants |
| --- | --- |
| <12 months | 200 + 200 Mothers |
| 1-10 years | 225 |
| 11-20 years | 225 |
| 21-30 years | 225* |
| 31-40 years | 225* |
| 41-50 years | 100 |
| 51-60 years | 100 |
| Total | 1500 |

*In addition to women randomly selected in each of these age groups, mothers of infant participants (recruited at birth) will also be recruited for participation. We anticipate that the majority of these women will be age 20-29 years with fewer in the age 30-39 years category.

**Sample size considerations**

   The sample sizes listed in the Study Design section will be sufficient for estimation of the proportions with adequate IgG antibody levels in each age category.  95% confidence intervals (CI) will be given for each estimate. The table below gives 95% CI widths based on the binomial distribution with sample sizes of 100, 200 and 225, for a range of binomial variances based on proportions of 0.50 (conservative) to 0.90 (likely). The calculations provide 90% probability that the given width or less will be achieved.

Table 2: Half-widths of 95% confidence intervals for the proportion of people with adequate IgG antibody levels within an age interval.

|  | **Proportion used for binomial variance estimation** | | | | |
| --- | --- | --- | --- | --- | --- |
| **Sample size** | **0.50** | **0.60** | **0.70** | **0.80** | **0.90** |
| **100** | 0.097 | 0.096 | 0.093 | 0.084 | 0.069 |
| **200** | 0.069 | 0.069 | 0.066 | 0.059 | 0.046 |
| **225** | 0.065 | 0.065 | 0.062 | 0.056 | 0.044 |

These calculations indicate that a 95% CI for the proportion with adequate measles antibodies will have width less than +/-0.10 with 90% probability for all the situations in Table 2. In a range of likely proportion estimates (close to 0.90), the 95% CI widths will be less than +/-0.069 for n=100, and less than +/-0.044 for n=225. These CI widths are small enough to yield useful interval estimates for public health planning.

**Methods for collecting specimens and data.**

This study will consist of an interview and collection of 2 ml blood sample collected via venipuncture for participants >= 1 year of age (and 1 ml blood sample via venipuncture in infants <1 year of age – and a cord blood sample from infants at birth) to be used for measles antibody testing.

**Study Population**

**Selection of the Study Population**

Chandigarh is a city and union Territory in northern India. Chandigarh had a population of 1,055,450 in the 2011 India Census. There are approximately 500 units call anganwadis located through Chandigarh. These anganwadis are units created to administer the Government of India’s [Integrated Child Development Services (ICDS)](http://en.wikipedia.org/wiki/Integrated_Child_Development_Services) scheme. The 500 anganwadis are categorised into three sets based on the ICDS services provided. The anganwadis are distributed throughout the population of Chandigarh comprising three categories; rural, urban and resettlement colonies (previously referred to as urban slum) It is expected that we will recruit participants from each of the three residential areas (rural, urban and resettlement colonies) in a proportion equivalent to the population distribution.

In total, 1500 participants between ages 0-60 years will be enrolled in the study. Informed consent will be obtained for each adult participant (i.e. >18 years). For children age 0-17 years parental consent will be obtained. For children age 8 to 17 years additional assent will be obtained, in addition to parental consent. All participants have the right to withdraw their participation at any time. More information about the informed consent process can be found in section 9 of this protocol.

A variety of individuals who live in the local community will assist the study staff in accessing and locating potential participants. These individuals include Anganwadi workers and community leaders who will assist study staff by providing access to records that facilitate the participant selection process by study staff, and by locating the selected participants. These personnel will not be involved in the direct selection process nor will they be involved in the participant recruitment process. Because they are prominent members of the community, many will inevitably have independent relationships with potential participants. Therefore, they will be reminded that only study staff will recruit participants. These community personnel will be paid a modest stipend of 300 Indian Rupees (INR) (approximately $5 US) per participant to compensate for time and expenses that result in assisting the study staff in accessing records, traveling to the community, and/or locating potential study participants.

***Recruitment Strategy***

From the communities served by Anganwadis in Chandigarh, we will select 30 communities using a stratified random sampling method with representative communities from rural, urban, and resettlement colonies. Participants will be selected from the 30 selected communities using two methods. For method 1, infants at birth and their mothers will be selected randomly from a list of infants born in each of the 30 selected communities. For method 2, participants age 1-60 years will be selected using a random household method described below.

**Selection method 1**

Pregnant women will be identified through anganwadi workers and local hospital/clinic records. These women will be recruited for study participation to begin at or near the time of delivery. Their newborn baby will be enrolled at birth; and then followed up at 3, 6, 9, and 12 months of age. Study staff will approach selected participants at their homes or at clinics (if they are known to be at the clinic for an appointment). This will be done in collaboration with the Anganwadi worker and local hospital and clinic staff who know the local residents and can assist in locating them. For every infant, the mothers will be recruited for participation as well. This methodology will yield 200 children and 200 mothers, making a total of 400 participants.

**Selection method 2**

For persons age 1 year and older, for each of the 30 randomly selected areas, study staff will seek the assistance of the Anganwadi worker to go to the community leader who maintains a list of all households in that community; Anganwadi workers will assist with introductions between the study team and the community leaders to facilitate the relationship.

Field staff will visit one Anganwadi each day and liaise with the Anganwadi worker. They will identify the list of houses covered by that Anganwadi worker. One household will be randomly selected from a list of households in the selected community. The study team will recruit the first participant from that household and then proceed to every fifth to tenth household (depending on the number of houses in the community), referred to as the next “eligible household” to recruit and enroll additional participants. Enrollment will be stratified by age group to ensure an even distribution across ages. If an eligible person in a given household declines to participate, the next eligible household will be approached. A total of 1100 people age 1-60 years will be enrolled from 30 communities using this methodology.

**Additional recruitment notes details**

- The homes of potential participants will be approached multiple times (up to 4) if a potential participant is not at home during the initial visit or if they request that the study staff returns at another time,
- Participants will be generally healthy and able to participate in normal activity.
- Minors will be included in this study in order to adequately characterize the epidemiology of measles in Chandīgarh, India. Parents or guardians may be present throughout the interview and specimen gathering.

The PI does not belong to an organization that is allowing access to its members or clients.

**Inclusion and Exclusion Criteria**

Inclusion criteria

- Individuals between age 0- 60 years.
- Individuals who are currently staying in Chandigarh, India

Exclusion criteria

- Individuals with a health condition that prohibits them from participating in normal daily activities
- Individuals with known hemophilia or other blood dyscrasias characterized by potential for excessive bleeding

Individual with acute febrile illness
